# Supplementary material for: Pathophysiological Significance of Neutrophilic Transfer RNA-Derived Small RNAs in Asymptomatic Moyamoya Disease
Source: Cells. 2021 May 1;10(5):1086. doi: 10.3390/cells10051086 (PMC8147334; doi:10.3390/cells10051086)

2D Structure	Local AU	Position

```
68                                    83
                  7mer-m8
5' - t a a t t t A T A A C C A G G G g - 3'  utr
                 |   :   | | | | | | |
3' - u g a u c U G G U G G U C C C u - 5'  tRF
    16 15 14 13           7 6 5 4 3 2
      3' Pairing             Seed
```

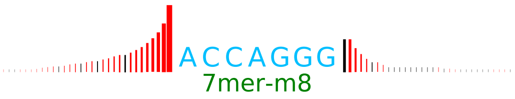

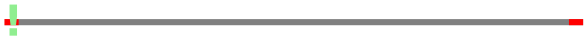

Supplement: Supplementary file 1 [file cells-10-01086-s001.zip › cells-1149161 3rd supplementary materials/Data Supplements/Data Supplement V.zip!/Data supplement 6-mRNAs targets of tsRNAs/tsRNA-Glu-CTC-003/Diagrams/tRF-Glu-CTC-003_vs_ENST00000374045(TNFSF15).pdf]
